# Facing the Challenges in the COVID-19 Pandemic Era: From Standard Treatments to the Umbilical Cord-Derived Mesenchymal Stromal Cells as a New Therapeutic Strategy

**DOI:** 10.3390/cells12121664

**Published:** 2023-06-19

**Authors:** Eleonora Russo, Simona Corrao, Francesca Di Gaudio, Giusi Alberti, Martin Caprnda, Peter Kubatka, Peter Kruzliak, Vitale Miceli, Pier Giulio Conaldi, Cesario Venturina Borlongan, Giampiero La Rocca

**Affiliations:** 1 Section of Histology and Embryology, Department of Biomedicine, Neurosciences and Advanced Diagnostics, University of Palermo, 90127 Palermo, Italy; eleonora.russo01@unipa.it (E.R.); giusi.alberti@unipa.it (G.A.); 2 Research Department, IRCCS ISMETT (Istituto Mediterraneo per per i Trapianti e Terapie Ad Alta Specializzazione), 90127 Palermo, Italy; corraosimona@libero.it (S.C.); vmiceli@ismett.edu (V.M.); pgconaldi@ismett.edu (P.G.C.); 3 PROMISE Department, University of Palermo, 90127 Palermo, Italy; francesca.digaudio@unipa.it; 4 1st Department of Internal Medicine, Faculty of Medicine, Comenius University, University Hospital Bratislava, 81499 Bratislava, Slovakia; martin.caprnda@fmed.uniba.sk; 5 Department of Medical Biology, Jessenius Faculty of Medicine, Comenius University in Bratislava, 03649 Martin, Slovakia; peter.kubatka@uniba.sk; 6 Research and Development Services, Pradlacka 18, 61300 Brno, Czech Republic; kruzliakpeter@gmail.com; 7 Department of Neurosurgery and Brain Repair, Morsani College of Medicine, University of South Florida, Tampa, FL 33612, USA

**Keywords:** COVID-19, SARS-CoV-2, Wharton’s jelly, mesenchymal stromal cells, umbilical-cord-derived mesenchymal stromal cells, extracellular vesicles, cytokine storm, inflammatory diseases, clinical trials, cell-based therapy, cell-free therapy

## Abstract

Coronavirus disease 2019 (COVID-19), the pandemic caused by severe acute respiratory syndrome coronavirus 2 (SARS-CoV-2), which counts more than 650 million cases and more than 6.6 million of deaths worldwide, affects the respiratory system with typical symptoms such as fever, cough, sore throat, acute respiratory distress syndrome (ARDS), and fatigue. Other nonpulmonary manifestations are related with abnormal inflammatory response, the “cytokine storm”, that could lead to a multiorgan disease and to death. Evolution of effective vaccines against SARS-CoV-2 provided multiple options to prevent the infection, but the treatment of the severe forms remains difficult to manage. The cytokine storm is usually counteracted with standard medical care and anti-inflammatory drugs, but researchers moved forward their studies on new strategies based on cell therapy approaches. The perinatal tissues, such as placental membranes, amniotic fluid, and umbilical cord derivatives, are enriched in mesenchymal stromal cells (MSCs) that exert a well-known anti-inflammatory role, immune response modulation, and tissue repair. In this review, we focused on umbilical-cord-derived MSCs (UC-MSCs) used in in vitro and in vivo studies in order to evaluate the weakening of the severe symptoms, and on recent clinical trials from different databases, supporting the favorable potential of UC-MSCs as therapeutic strategy.

## 1. Introduction

After the three worldwide influenza outbreaks in the 20th century that were named in relation with the cite of origin (Spanish, 1918; Asian, 1957; Hong Kong, 1968), characterized by the infection of three different subtypes of influenza A virus (H1N1, H2N2, and H3N2, respectively) [1], the world recently faced the ongoing pandemic at the end of 2019, since Chinese health authorities informed of an outbreak of pneumonia of unknown etiology in Wuhan City, Hubei Province, on 31 December 2019 [2,3,4,5]. World Health Organization (WHO) established this as a public health emergency of international concern on 30 January 2020 [6]. The unknown origin was, thereafter, recognized as a possible zoonotic transmission, starting from bat and pangolin coronaviruses, spreading across other intermediate host species [7]. On 11 February 2020, the International Committee on Taxonomy of Viruses (ICTV) named this virus “severe acute respiratory syndrome coronavirus 2 (SARS-CoV-2)” and the WHO named the related disease “Coronavirus Infectious Disease (COVID)-19” [8]. The SARS-CoV-2 genome consists of 29,903 nucleotides, and a phylogenetic analysis suggested that the virus is closely linked (89.1% nucleotide similarity) to a group of SARS-like coronaviruses (genus Betacoronavirus, subgenus Sarbecovirus) that had been reportedly found in animal hosts such as bats in China [9,10,11,12]. Following a global infection of SARS-CoV-2, COVID-19 was declared as a pandemic by WHO on 11 March 2020 [13]. Several clinical symptoms of COVID-19 are common to severe acute respiratory syndrome coronavirus (SARS-CoV) and Middle East respiratory syndrome coronavirus (MERS-CoV), such as fever, nonproductive cough, dyspnea, myalgia, fatigue, normal or decreased leukocyte counts, and radiographic evidence of pneumonia [2,14]. However, as described by WHO, the progression of the disease on vulnerable populations, such as pediatric patients, older people, and pregnant women, contributes to respiratory complication, multiorgan failure, the need for mechanical ventilation, and the admission to intensive care unit (ICU), where critical stage of disease could be potentially lethal [15]. According to the WHO data on 21 December 2022, the number of confirmed cases reached more than 650 million worldwide, with more than 6.6 million of counted deaths. These numbers are continuously updated on the dashboard of WHO (website: https://covid19.who.int/, accessed on 21 December 2022). Standard medical care for treating COVID-19 symptoms (mainly anti-inflammatory drugs) still awaits specific tools in order to counteract the most severe forms, which could lead to death. Research is moving along and it is supported by an enormous deployment of forces in terms of discovering new strategies and drugs. Importantly, the introduction of vaccines has reduced the spread of COVID-19 infection, even though the rapid increase in viral variants has reduced the effectiveness of vaccination, leading to the need for a booster dose against new viral strains. In addition, pharmacological approaches have been tested to treating COVID-19 patients, including anti-inflammatory and antiviral drugs. As a result, the damage caused by SARS-CoV-2 infection remains a long-term concern. Therapies based on the use of exogenous cells could represent an alternative and profitable strategy [16]. It is now known that mesenchymal stromal cells (MSCs) and their extracellular vesicles (EVs) represent a gold standard in regenerative medicine for several reasons, such as multipotent differentiation potential, immunomodulation and anti-inflammatory properties, mitochondrial transfer, and promotion of endogenous repair mechanisms [17]. MSCs can be isolated by adult tissues such as bone marrow (BM-MSCs) or adipose tissue (AT-MSCs), but also from perinatal tissues, including placenta (PL-MSCs), umbilical cord (UC-MSCs), umbilical cord blood (UCB-MSCs), and amniotic fluid (AF-MSCs). In recent years, consistent research has been carried out on EVs secreted by MSCs, which are enriched in proteins, lipids, and nucleic acids. Moreover, EVs can be used as a vehicle for drug delivery. Interestingly, it has been observed that UC-MSCs-derived EVs present a therapeutic potential for the treatment of different diseases, including COVID-19 [18,19,20]. Taking into account all these considerations, we focused this review on the recent in vitro and in vivo research in addition to the current clinical studies that involve the use of UC-MSCs in the treatment of the inflammatory status related to SARS-CoV-2 infection.

## 2. SARS-CoV-2 General Features and Mechanism of Infection

### 2.1. Genome, Structure, and the Variants of Concern behind the High Transmissibility

**Figure 1:**
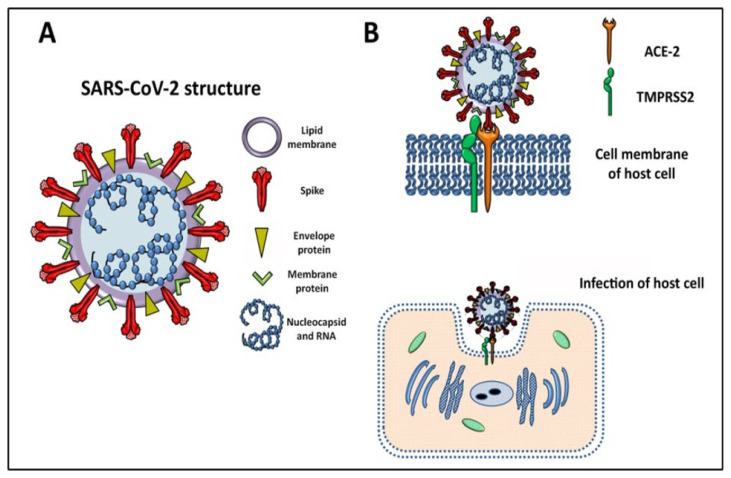
(**A**) Schematic description of SARS-CoV-2 structures is characterized by the RNA genome containing viral information associated with nucleocapsid, while structural spike, envelope, and membrane proteins are anchored in the lipid membrane. (**B**) The infection of the host cell by SARS-CoV-2 is dependent on unique interaction between spike protein and the angiotensin-converting enzyme 2 (ACE-2). The presence of transmembrane serine protease 2 (TMPRSS2) guarantees the activation of viral fusion and internalization of the virus for the subsequent viral genome replication.

The first SARS-CoV-2 genome sequence was published on the community online resource virologial.org on 10 January 2020 (Wuhan-Hu-1, GenBank accession number MN908947) [21]. Based on the viral sequence database Global Initiative on Sharing All Influenza Data (GISAID, https://www.gisaid.org/, accessed on 21 December 2022), more than 12 million viral genomic sequences have been submitted. SARS-CoV-2 is an enveloped virus consisting of a single-stranded positive-sense RNA on which specific genes (5′ to 3′) are transduced for replicase ORF1a/b, and four structural components: spike (S), envelope, membrane, and nucleocapsid proteins [11]. A schematic structure of SARS-CoV-2 is depicted in Figure 1A, showing the proteins anchored to the lipid membrane. Similar to other SARS-CoVs, infected droplets or aerosols first target the respiratory mucosal epithelium, initiating viral infection, even if the reason for the fast spread of the infection worldwide and both asymptomatic and symptomatic patients demonstrated high levels of viral load in the lower-airway lung cells [10,12,22,23].

As expected, the diffusion of the virus and the increase of infections drive the generation of new mutations in the viral genome. Different mutations in SARS-CoV-2 S protein, nucleocapsid protein, and ORF3a have been found worldwide [24]. A phylogenetic network analysis conducted by Forster et al. showed three major variants of SARS-CoV-2 globally, named A, B, and C, diffused in United States and Australia, East Asia, and Europe, respectively [25]. Among the four structural genes, the S gene revealed a series of mutations which is, in turn, related with generation of variants of the virus in the S protein. Notably, the first SARS-CoV-2 variant of concern was identified on 16 December 2020, in the UK, consisting of 17 changes or mutations [26]. Variations occurring in the SARS-CoV-2 genome resulted, in some cases, in variants of concern, among which the most known are Alpha (B.1.1.7), Beta (B.1.351), Gamma (P1), Delta (B.1.617.2), and Omicron (B.1.1.529), giving rise to differences in symptoms, pathogenicity, viral load, increased rate in transmissibility, and reduced effectiveness of current diagnostic methods, therapies, and vaccines [27,28,29]. Even early and accurate detection of SARS-CoV-2 with efficient laboratory diagnostic tests has been thought necessary for facilitating public health interventions and interrupting the transmission chain [30,31].

### 2.2. Mechanism of Infection SARS-CoV-2, the Cytokine Storm, and Pathogenesis of COVID-19

The progression of COVID-19 in lung tissue on deceased donors revealed that viral RNAs were enriched in mononuclear phagocytic and endothelial lung cells which induced specific host response, while spatial analysis distinguished inflammatory host responses in lung regions with and without viral RNA [32]. As mentioned above, the SARS-CoV-2 S protein is necessary for infection, and this is related to its binding on the angiotensin-converting enzyme (ACE)-2 receptor expressed by host cells, and the receptor-mediated virus entry depends on a serine protease, transmembrane serine protease 2 (TMPRSS2) (Figure 1B) [9,33,34,35]. An scRNA-seq analysis of barrier tissues and model organisms aimed to identify the initial cellular targets of SARS-CoV-2 infection. The authors assessed that ACE-2 expression was a characteristic of certain cell types, such as type II pneumocytes in the lung [36,37], gut enterocytes, and goblet secretory cells of the nasal mucosa. On the other hand, the ACE-2/TMPRSS2 co-expression in respiratory tissues was limited to a rare subset of epithelial cells [32]. Moreover, ACE-2 has been identified in several tissues such as lungs, kidneys, heart, endothelial, and intestinal cells, as well as in liver, pancreas, reproductive tract, and central nervous system (CNS) [38,39,40,41,42]. For this reason, COVID-19 patients also present nonpulmonary or atypical manifestations, including headache, dizziness, olfactory and taste disorders, nausea, abdominal pain, vomiting, and diarrhea [39]. Not all of the cells express ACE-2. For example, bone marrow, lymph nodes, thymus, spleen, and immune cells, such as T and B lymphocytes and macrophages, are negative for ACE-2 [43]. Nevertheless, other receptors interacting with S protein, expressed even by ACE-2-negative cells, have been proposed for the entry of SARS-CoV-2 in cells expressing C-type leptin receptors (CLRs), such as CD209L (also known as L-SIGN) and CD209 (also known as DC-SIGN) [44,45]. The DC-SIGN/CD209 (dendritic-cell-specific intercellular adhesion molecule-3-grabbing nonintegrin) and the other CLRs can regulate immune responses via Toll-like receptors (TLRs), and TLR2 cooperates with the monocyte surface molecule CD14 in response to viral infection, leading to the activation of nuclear factor kappa-light-chain-enhancer of activated B cells (NF-kB), inducing the production of inflammatory cytokines.

The structure of the SARS-CoV-2 S protein is similar to the SARS one, but its ACE-2-binding affinity is 10 to 20 times higher, and this could be a reason for the higher transmissibility of SARS-CoV-2 [46]. TMPRSS2 cleaves the S protein activating the virus to fuse with host lipid bilayer membrane, followed by the deposition and replication of the viral RNA genome into the host cell. The viral double-stranded RNA (dsRNA), created during replication, generates intermediates that can activate the cytoplasmic innate immune pathway, initiating a signaling cascade that leads to the production of type I and type III interferons (IFNs), the first antiviral function [47].

After internalization, the replication of the virus inside the host lung cells is followed by increased infiltration and activation of macrophages, as discovered in biopsy or autopsy specimens from patients with SARS-CoV-2 infection [48]. It has been established that the early response phase is characterized by the innate production of cytokines and the induction of emergency granulopoiesis, leading to the mobilization of neutrophils and monocytes [49,50]. Over 80% of COVID-19 patients present lymphopenia and an increased neutrophil–lymphocyte ratio [51], corresponding to low percentages of CD3^+^ T cells in peripheral blood [52]. All the events that follow virus entry lead to an imbalanced innate and adaptive host response, defined by low levels of type I and III interferons (IFNs), elevated chemokines, and high expression of interleukin (IL)-6, inducing a reduction of innate antiviral defenses [53], and generating the so-called “cytokine storm”, one of the main mechanisms for ARDS, which is the main cause of death in COVID-19 patients. The type II alveolar pneumocytes produce an innate proinflammatory response to SARS-CoV-2 infection, secreting proinflammatory cytokines such as tumor necrosis factor (TNF)-α, IL-6, IL-1β, monocyte chemoattractant protein-1 (MCP-1), and granulocyte-macrophage colony-stimulating factor (GM-CSF) [36]. In addition, SARS-CoV-2 induces a rapid increase of pathogenic T helper (Th)1 lymphocytes expressing GM-CSF and IFN-γ. This is associated with an increased proliferation of inflammatory CD14^+^ CD16^+^ intermediate monocytes releasing both GM-CSF and IL-6, TNF-α, and IL-1β, thus contributing to cytokine storm [36,54]. Based on new nomenclature, the major population of human monocytes (90%) is described as the “classical” monocytes, which feature high levels of CD14 but are negative for CD16 (CD14^++^CD16^−^, or CD14^+^CD16^−^). The remaining group of human monocytes (10%) is further subdivided into the “intermediate” subset, in which CD16 is expressed alongside CD14 (CD14^++^CD16^+^, or CD14^+^CD16^+^), and the “nonclassical” subset, which features high expression of CD16 but a lower positivity to CD14 (CD14^+^CD16^++^ or CD14^dim^CD16^+^) [55]. Classical and intermediate monocytes are involved in inflammatory responses, while nonclassical ones are responsible for “patrolling behavior”, crawling the endothelium and supporting blood vessels integrity and antiviral functions [56]. The classical and intermediate monocytes observed in COVID-19 patients, including specimens such as plasma and bronchoalveolar lavage fluid, featured CD169^+^ expression [57]. In the lungs, the presence of macrophages is at multiple levels, i.e., alveolar and interstitial macrophages. Both alveolar and interstitial macrophages can be in two functional phenotypes, proinflammatory M1 macrophages and anti-inflammatory M2 macrophages, and since they express ACE-2, they produce proinflammatory cytokines/chemokines, leading to exacerbation of lung infection, resulting in ARDS. Thereafter, macrophages can migrate out of lungs contributing to systemic inflammations [58].

There is a natural temporal arc from induction to resolution of an immune response, with magnitude and duration that is finely orchestrated and balanced. Any disruption of this arc can lead to hyperimmune responses, or a delay in the resolution phase [49]. The impaired inflammatory response in COVID-19 patients may be due to a singular low expression of type I and III IFNs, resulting in reduction of antiviral defense, associated with elevated NF-kB-induced chemokines, leading to leukocytes recruitment and high expression of proinflammatory IL-6 [48]. Nevertheless, as described by Leisman and coworkers, the systemic inflammatory profile of COVID-19 patients was different compared with those having non-COVID-19 ARDS, sepsis, or chimeric antigen receptor T cell (CAR-T)-induced cytokine release syndrome. Several noncytokine biomarkers (including D-dimer, C-reactive protein, and ferritin) were elevated to a comparable or higher amount in patients with COVID-19 than in patients with the other disorders [59]. COVID-19 patients showed increased levels of D-dimer, fibrinogen, and profibrotic molecules such as platelet-derived growth factor-BB (PDGF-BB) and matrix metalloproteinase (MMPs) [60,61,62]. The disease duration in ICU patients positively correlated with an extremely increased of plasma protein levels of IL-13, IL-1β, GM-CSF, and the vascular endothelial growth factor (VEGF) beyond day 20 after symptom onset [63]. Ackermann et al. found a significant perturbation of angioarchitecture in the postmortem lung of seven patients who died due to COVID-19, showing variations in the caliber of the capillaries which exhibit cylindrical microstructures in the lumina and describing, for the first time, intussusceptive angiogenesis in the pathogenesis of COVID-19 [64]. The alveoli environment is also impaired with activation of endothelial cell death, platelet activation, exposure of extracellular matrix (ECM), the presence of active tissue factor, and extrinsic and intrinsic coagulation [47]. The formation of fibrin clot and platelet activation increase the risk of death [65].

### 2.3. The Role of Angiotensin II in Tissue Homeostasis Disruption and Multiorgan Failure

**Figure 2:**
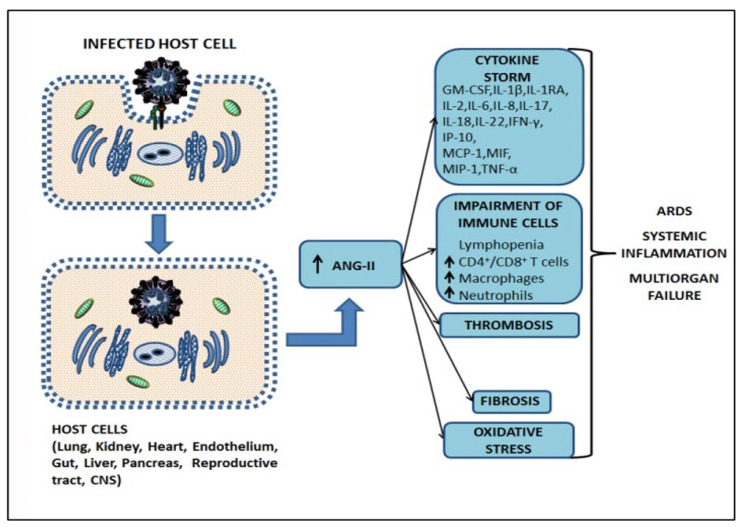
Schematic representation of the different aspects of COVID-19 pathogenesis. SARS-CoV-2 binds to the host cell, inducing ACE-2 endocytosis. The reduction of ACE-2 on cell surface determines an increase of serum angiotensin II (ANG-II,) triggering cytokine storm, impairment of immune cells, thrombosis, fibrosis, and oxidative stress contributing to the ARDS, systemic inflammation, and multiorgan failure. GM-CSF: Granulocyte-macrophage colony-stimulating factor; IL-: Interleukin (IL-1β, IL-1RA, IL-2, IL-6, IL-8, IL-17, IL-18, IL-22); IFN-γ: Interferon γ; IP-10: IFN-γ-induced protein 10; MCP-1: Monocyte chemoattractant protein-1; MIF: Macrophage migration inhibitory factor; MIP-1: Macrophage inflammatory protein 1; TNF-α: Tumor necrosis factor α.

The ACE-2 receptor is also a negative regulator of the renin–angiotensin–aldosterone system (RAAS) and functionally lowers blood pressure by catalyzing angiotensin (ANG)-II into the vasodilator ANG (1–7) [37]. When ACE-2 is endocytosed together with SARS-CoV-2, this results in the reduction of ACE-2 on the cell surface and a consequent increase of serum ANG-II, leading to hypertension [66]. ANG-II has proinflammatory properties, including the increase of IL-6 through the activation of NF-kB and signal transducer and activator of transcription 3 (STAT3) pathways [67]. TNF-α and NF-kB trigger oxidative stress, and the interaction of ANG-II with angiotensin type 1 receptor (AT1R) activates NADPH oxidase (NOX), causes a reduction of nitric oxide (NO) bioavailability, and causes redox imbalance at mitochondrial level, determining the excess in reactive oxygen species (ROS), leading to vascular endothelial damage and prothrombotic events [68,69]. In addition, ANG-II causes a disintegrin and metalloproteinase 17 (ADAM17)-mediated shedding and activation of TNF-α-signaling [70]. It is involved in the increased expression of connective tissue growth factor (CTGF), a profibrotic factor involved in fibroblast proliferation, cellular adhesion, ECM deposition, and activation of transforming growth factor β1 (TGF-β1) signaling that stimulates myofibroblasts differentiation and ECM biosynthesis, along with the preservation of ECM proteins by regulation of MMPs and tissue inhibitors of metalloproteinases (TIMPs) [71,72]. ANG-II is also involved in reactive oxygen species (ROS) generation, resulting in endoplasmic reticulum stress, mitochondrial dysfunction, and vascular oxidative stress [67]. Consequently, a central role is played by the ANG-II, which increase in SARS-CoV-2 infection and lung dysfunction [73,74]. The increased level of ANG-II triggered by SARS-CoV-2 infection can result, in turn, in vasoconstriction as well as vasculopathy, coagulopathy, inflammation, and profibrotic effects, due to its effects in ECM remodeling, leading to multiorgan failure [66,71,75,76]. A descriptive sequence from host cell infection to severe COVID-19-related symptoms is shown in Figure 2.

## 3. From the First State of Emergency to the Current Standard Treatments of COVID-19 Patients

### 3.1. Social Distancing, Face Mask Wearing, and Convalescent Plasma

At the first signs of pandemic characteristics, in the absence of immediate protocols for treatment, emergency lockdowns, physical distancing, and face mask wearing have been mandatory across the world, since they have been thought to be the best first approach to prevent or reduce the rate of infection, but these have affected the health, business, and other aspects of daily life throughout societies. In a systematic review and meta-analysis, the authors suggested that transmission of virus was lower when maintaining a physical distancing of 1 m or more [77]. As confirmation, a large prospective U.S. cohort study (198,077 participants), showed that people living in communities with the greatest social distancing had a 31% lower risk of predicted COVID-19 compared with those living in communities with poor social distancing. It has to be noted that the use of face masks was associated with a 62% reduced risk of predicted COVID-19 even among individuals who lived in low social distancing conditions [78]. Nevertheless, the social behavior alone was not able to avoid the onset of pandemic, the increase of patients with moderate-to-severe form needing hospitalization, and the mechanical ventilation support for those with severe form of respiratory failure. Up to date, there is not a definitive therapeutic strategy for COVID-19 whose efficacy has been proven over the attempted solutions.

Even if it was seen as a promising strategy, the administration of plasma from convalescent patients, the hyperimmune plasma, did not exert any significant reduction in mortality and had minor impact on clinical improvement in individuals with moderate to severe disease [79]. A Chinese meta-analysis study [80] described that, in 32 randomized controlled trials and 21,478 patients, convalescent plasma therapy was not associated with significantly reduced 28-day mortality in COVID-19 patients, and it was not related to improvements in other survival outcomes (length of hospitalization, time without respiratory support, risk of symptoms progression, and requirement of mechanical ventilation). Moreover, in terms of safety, the treatment presented a trend with no statistical significance of higher incidence of adverse events, suggesting that the treatment could be recommended only in the context of clinical trials for severe COVID-19 patients, due to limited suppressive effect on inflammation and no significantly improved clinical outcomes [80].

### 3.2. IL-6 Receptor Blockers, Monoclonal Antibodies, and Antiviral Agents: The Recommendation of the WHO

Regarding drugs administration and therapeutics to treat COVID-19 patients, the WHO drafted and constantly updates living guidelines. Since COVID-19 is primarily related to increased IL-6 levels, WHO recommends both corticosteroids and IL-6 receptor blockers in patients with severe and critical COVID-19. However, corticosteroids, such as dexamethasone, are not recommended for nonsevere COVID-19 patients, but only for patients undergoing septic shock, or in critical cases [81,82]. Colchicine is not recommended, except in clinical trials, while other anti-inflammatory drugs (fluvoxamine or budesonide) did not provide sufficient evidence [83]. The potential role of IL-6 in COVID-19 pneumonia led to testing anti IL-6 receptor blockers, such as Tocilizumab and Sarilumab, humanized monoclonal antibodies used for other inflammatory diseases [84]. It has been proven by clinical studies that Tocilizumab and Sarilumab are both effective, as compared with the current standard of care, with reduced death rates in COVID-19 patients featuring severe illness, rapid deterioration, and increasing oxygen needs, and who had a significant inflammatory response [85,86]. Controversial results came from other studies: in a randomized trial involving hospitalized patients with severe COVID-19 pneumonia, the use of tocilizumab did not result in significant improvement of clinical status or lower mortality compared to placebo at 28 days [87]. In contrast, a prospective meta-analysis of clinical trials in patients hospitalized for COVID-19, conducted by the WHO Rapid Evidence Appraisal for COVID-19 Therapies (REACT) Working Group, suggested that administration of IL-6 antagonists was associated with lower 28-day all-cause mortality [88]. Moreover, because of emergency situations, the heterogeneity of population, and different dosages administered, the first data on the safety and effectiveness of tocilizumab in severe COVID-19 were retrieved from observational retrospective studies [89]. Additionally, IL-6 receptor blockers cost is still a challenge. Up to date, the latest version of “Therapeutics and COVID-19: living guideline” (September 2022) regarding other monoclonal antibodies, such as casirivimab and imdevimab, demonstrated a lack of efficacy against the Omicron BA.1 variant. Therefore, casirivimab–imdevimab administration is no longer recommended for COVID-19 treatment except in cases where infection with a SARS-CoV-2 variant (such as Delta) is confirmed at sequencing level [90].

Other drugs with antiviral and anti-inflammatory mechanism of actions (such as remdesivir, lopinavir/ritonavir, chloroquine, and hydroxychloroquine) did not show evidence in reducing mortality or need for mechanical ventilation, and there was a risk of adverse events including diarrhea, nausea, and vomiting leading to the risk of hypovolemia, hypotension, and acute kidney injury [90]. In addition, convergent evolution on Omicron S protein has recently generated sublineages (such as the so called “Centaurus”, “Cerberus”, and “Gryphon”) which are able to escape anti-Spike monoclonal antibody therapies [91,92]. Fortunately, antiviral agents, such as remdesivir, molnupiravir, and nirmatrelvir, have been recently demonstrated as efficacious against both BQ.1.1 (“Cerberus”) and XBB (“Gryphon”) in vitro [93].

### 3.3. Vital Support: Prone Positioning, Mechanical Ventilation, and Extracorporeal Membrane Oxygenation (ECMO)

In individuals with low response to drugs, with a refractory respiratory failure and severe ARDS, lung function is seriously compromised, leading to severe impairment of gas exchange, hypoxemia, and impaired CO_2_ clearance in the alveolar space [94]. Therefore, in-hospital care is needed. Prone positioning for nonintubated patients has been widely applied and studied in COVID-19 patients. The results demonstrated that it exerted a reduction of lungs compression, since inducing a different gravitational-dependent redistribution of fluids. However, many questions are still unanswered, and randomized trials are ongoing in order to assess the clinical benefits of prone positioning in the management of COVID-19 patients [95].

Physicians need also to be able to critically evaluate the right conditions for giving supplemental oxygen, mechanical ventilation, and endotracheal intubation, due to the risks related with [96]. Mechanical ventilation is a double-edged sword because, on one hand, it is the usual treatment to support the respiration during ARDS, while, on the other, it is leading to ventilator-induced lung injury (VILI), causing lung fibrosis [97]. Moreover, ARDS shows that the deposition of additional ECM can result in a fibrotic remodeling of the lungs with related collapsed alveoli that need specific treatments for restoring alveolar space, such as mechanical ventilation PEEP (positive end expiratory pressure) [98].

Another invasive technique for blood reoxygenation is the venovenous extracorporeal membrane oxygenation (VV-ECMO), which can rest the lung functions, giving them time to recover, and it was revealed that ECMO is more effective in patients with more severe hypoxemia, even if the survival rate was not better compared with patients without ECMO [99]. Furthermore, ECMO could be beneficial but with higher risk of complication and mortality compared with influenza [100,101].

## 4. The Anti-SARS-CoV-2 Vaccines

In parallel with the definition of drug administration protocols, prevention through vaccination is the most effective way to reduce the spread of SARS-CoV-2 infection and the severe form of the disease. Based on “COVID-19—Landscape of novel coronavirus candidate vaccine development worldwide” distributed by the WHO (last publication, on 3 January 2023, available as a summary table) [102], more than 370 vaccines have been developed against SARS-CoV-2: 199 in preclinical and 176 in clinical development. The candidates in clinical development are 11 at phase IV, 50 at phase III, 15 at phase II/III, 14 at phase II, 31 at phase I/II, and 53 at phase I [102]. In particular, the mRNA-based vaccines BNT162b2 “Comirnaty” (produced by Pfizer-BioNTech) and mRNA-1273 Spikevax (produced by Moderna) have an efficacy rate near 95% to prevent COVID-19 disease and surprisingly with comparable outcomes [103,104,105], even considering the long-term effectiveness [106,107]. The effect has been proven also in frail individuals, such as immunocompromised solid organ transplant recipients, who featured improvements in both humoral and cellular-specific immune responses against the SARS-CoV-2 virus [108]. Another m-RNA-based vaccine is CVnCoV (CureVac, developed by CureVac N.V. and the Coalition for Epidemic Preparedness Innovations), while other vaccines are based on (i) viral vector (nonreplicating) (VVnr), such as ChAdOx1 (AstraZeneca), Ad26.COV2.S (Janssen, Johnson & Johnson), and Gam-COVID-Vac (the Sputnik V, developed by the Federal State Budgetary Institution N.F. Gamaleya Federal Research Centre for Epidemiology and Microbiology of the Ministry of Health of the Russian Federation); (ii) inactivated virus, such as CoronaVac (Sinovac), WIBP-CorV and BBIBP-CorV, BBV152 (COVILO) (Sinopharm), and BBV152 (Covaxin, Bharat Biotech); (iii) protein subunit, such as NVX-CoV2373 (Nuvaxovid, Novavax) and FINLAY-FR-2 (Soberana 02, produced by the Finlay Institute, a Cuban epidemiological research institute). It was described that there is high-certainty evidence that these vaccines reduce severe or critical disease, even if with important differences in vaccine efficacy, with little or no difference between most vaccines and placebo for serious adverse events (SAEs) [105]. Lower percentages of vaccine candidates are based on viral vector (replicating) (VVr), virus-like particle, VVr + antigen-presenting cell, live attenuated virus, VVnr + antigen-presenting cell, and bacterial antigen–spore expression vector [102]. The current vaccines become less effective the more the mutations occur, as in the case of seasonal flu vaccine, which is adjusted accordingly every year. Moreover, emerging evidence reported waning immunity after 6 months of completed vaccination [109]. Therefore, these issues drove the need to offer a booster dose of vaccine to restore the effectiveness of vaccination [110]. Another aspect worthy of attention is that the availability in large quantities and an effective campaign of vaccination of people are needed in lot of countries to reach a significant level of immunity.

However, despite the prevention of the severe form of the illness with vaccination, some difficulty in completing the vaccination campaign, placing an important pressure on ICUs for treating severe and critical stages of COVID-19 and ARDS in elderly patients, immunocompromised patients, and those with comorbidities (chronic obstructive pulmonary disease, cardiovascular diseases, diabetes), together with the risks of side effects after drug administration [90], multiorgan failure, and tissue damages, have made it necessary to continuously develop new therapeutic strategies. New approaches are recently focusing on the use of cells with high anti-inflammatory, regeneration, and tissue repair potency, such as the mesenchymal stromal cells (MSCs).

## 5. Characteristics of UC-MSCs in In Vitro and Preclinical Experimental Evidence Supporting Anti-Inflammatory, Immunomodulation, and Therapeutic Potential

### 5.1. Adult and Perinatal MSCs: General Features

Mesenchymal stromal cells (MSCs), which derive from the inner mass of the blastocyst, have a high capacity to self-renew, have fibroblastic-like shape when cultured in plastic surface, can differentiate into mesodermal derivatives such as osteoblasts, chondrocytes, and adipocytes, and show phenotype and characteristics in accordance with the minimal criteria of the International Society for Cellular Therapy (ISCT) [111]. They have, therefore, shed a new light on treatment of patients suffering from diseases and disorders that do not yet have a definite cure, and have a long history since their discovery to therapy applications [112]. MSCs are present in almost all post-natal/adult organs, i.e., bone marrow [113,114,115], adipose tissue [116,117], dental pulp [118,119], endometrium [120,121], menstrual blood [122,123], peripheral blood [124], salivary gland [125,126], skin and foreskin [127,128,129,130], synovial fluid [131,132], muscle [133,134,135], corneal stroma [136,137], heart [138,139], and lung [140]. Promising sources of MSCs are represented by the extraembryonic/perinatal tissues [141], among which there are the placenta, the chorionic and amniotic membranes [142,143,144], amniotic fluid [145], umbilical cord blood [146], and umbilical cord stroma [147]. Moreover, since MSCs derived from perinatal, as well as adipose tissue (AT-MSCs) and bone marrow (BM-MSCs), do not express ACE2 and TMPRSS2, this demonstrates that they are not permissive to SARS-CoV-2 infection, increasing the interest in the use of MSCs as potential therapy for COVID-19 [148]. However, the methods for obtaining adult tissues are invasive, and the yield of cells gained after isolation is scarce (e.g., 3.5 × 10^5^ to 1 × 10^6^ in 1 g of adipose tissue and from 500 to 5 × 10^4^ from 1 g of bone marrow aspirate [149]). Perinatal tissues provide an interesting source of MSCs as they are usually wasted after birth and, therefore, the collection procedures are without risks for the donor and ethical issues [150]. Importantly, umbilical cord (UC) matrix is a better source of MSCs, in terms of yields, than the umbilical cord blood and adult tissues [151,152,153]. Furthermore, the adult MSCs displayed higher susceptibility to senescence and oxidative stress along with the passaging in culture compared to perinatal MSCs [154].

### 5.2. UC-MSCs Properties: Multilineage Differentiation, Immune Tolerance, Angiogenesis/Wound Healing, Matrix Remodeling, and Resistance to Hypoxia

Multilineage differentiation properties—The UC is consisted of two arteries and one vein included in a connective tissue called “Wharton’s jelly” (WJ), mainly composed of sponge-like structure woven with collagen fibers, proteoglycans, and embedded MSCs, and an outer layer of amniotic epithelium [147,155,156]. Our group and others focused research on molecular characterization of UC-MSCs, demonstrating that these cells express CD10, CD13, CD29, CD44, CD54, CD73, CD90, CD105, Stro-1, MHC class I (classical HLA-A, -B and -C), mesenchymal markers (vimentin, α-SMA), neuroectodermal markers (Nestin, NSE, GFAP), and early endoderaml markers (GATA-4,-5,-6, HNF4, cytokeratin-8,-18,-19), and lack the major costimulatory molecules responsible for T cell activation, specifically B7-1 (CD80) and B7-2 (CD86), hematopoietic and endothelial markers CD14, CD19, CD31, CD34, CD38, CD45, CD66b, CD80, CD86, CD106, and CD133, [147,157,158,159,160]. UC-MSCs multipotency is formally demonstrated by their in vitro differentiation capability towards cell types of mesodermal origin (chondrocytes, adipocytes, osteoblasts, odontoblast-like cells, dermal fibroblasts, smooth muscle cells, skeletal muscle cells, cardiomyocytes) and endodermal lineages (hepatocyte-like cells, pancreatic endocrine cells), as well as ectodermal and neuroectodermal (sweat gland cells, oligodendrocytes, and dopaminergic neurons) [147,157,161,162,163,164,165,166,167,168,169,170,171,172,173,174]. UC-MSCs also feature “primitive stemness” properties due to their close relation with the embryologic phase, also maintaining the length of telomeric ends even after around 60 population doublings, and having no chromosomal mutation acquisition [157].

Immune tolerance and anti-inflammatory properties—UC-MSCs exert immunomodulation properties [163,175,176], even related to the expression and release of specific factors, such as the nonclassical HLA class I antigen, HLA-G [157,177], HLA-E, CD276/B7-H3, leukemia inhibitory factor (LIF), indolamine 2,3-dioxygenase-1 (IDO-1), galectin-1 (Gal-1), and heat shock protein 10/Early Pregnancy Factor (HSP10/EPF), being able to modulate or inhibit lymphocyte proliferation [175]. These factors are involved in tolerogenic processes occurring at the fetal–maternal interface [178,179,180,181,182,183], permitting, in turn, the semi-allogeneic embryo to escape surveillance of the maternal immune system. Specifically, HLA-G is an inhibitory molecule involved in immune tolerance and exerts its inhibitory functions interacting with inhibitory receptors Ig-like transcript (ILT) receptors, such as ILT-2, ILT-3, and ILT-4, and killer cell immunoglobulin-like receptor (KIR), two Ig domains, and long cytoplasmic tail 4, KIR2DL4, differentially expressed by NK, T, and antigen-presenting cells [184,185]. Its expression is enhanced by Gal-1 [183]. HLA-G also interacts with leukocyte immunoglobulin-like receptor subfamily B member 1 (LILRB1) expressed by CD56^bright^CD16^−^ natural killer (NK) cells that are enriched in the uterus during pregnancy, are poorly cytotoxic, and produce low amounts of IFN-γ as compared with peripheral blood CD56^dim^CD16^+^ NK cells [160]. HLA-E downregulates the immune response at the fetal–maternal interface, cooperating with classical HLA class I molecules in order to protect target cells from NK-cells-mediated cytotoxicity, interacting with CD94/NKG2A receptor [178]. CD276/B7-H3 expressed by UC-MSCs is able to inhibit T cell proliferation in a mixed lymphocyte reaction assay [175], and it is known to promote the survival of Th2 T cells over Th1 ones, together with indirect, noncontact-dependent mechanism mediated by IDO-1 and Gal-1 [186,187]. IDO-1 deprives effector T cells from tryptophan, reducing their proliferation and promoting apoptosis through O_2_ free radicals production, as well as inducing tolerogenic Tregs through interactions between naïve T cells and the products of tryptophan metabolism [188].

Studies show that LIF induces proliferation of CD4^+^ Foxp3^+^ Tregs, an anti-inflammatory phenotype in macrophages. In addition, it promotes survival of neurons and oligodendrocytes, and stimulates neurite outgrowth, all features that contribute to improving the clinical condition of experimental autoimmune encephalitis (EAE), a reliable mice model of human multiple sclerosis [189,190]. LIF production may play an important role in homeostasis and repair in the human lung tissues after induction by cytokines [191], and it could potentially support the use of UC-MSCs for restoring lung functions altered by SARS-CoV2 infection.

Lastly, HSP10, commonly known as a heat shock protein of 10 KDa, mainly expressed in the inner membrane of mitochondria, is also known as early pregnancy factor (EPF), discovered in the 1970s as a factor released during pregnancy in the serum of within 24 h after fertilization, preventing T cell-rosette formation and reduction of proinflammatory cytokines release, such as TNF-α (through interaction with TLR4 expressed by macrophages) (see in [192]). We found that Hsp10 was expressed by UC-MSCs both in vitro and in situ [175]. We were also the first to describe, in an in vitro COPD model, that Hsp10 showed a translocation from the cytoplasm to the nucleus, after exposure of the lung fibroblast cell line to cigarette smoke extract, but without direct interaction with DNA [193], thus suggesting a possible role of Hsp10 in gene expression modulation through chaperoning mechanism in response to pulmonary oxidative stress.

Although the underlying mechanism is still unknown, UC-MSCs secrete different prostanoids, such as PGD2, PGF2a, and PGE2, and several molecules, including IL-1R antagonist, IL-6, IL-10, M-CSF, VEGF, TGF-β1, and B7-H4, which could contribute to the differentiation of M2 macrophages [160]. UC-MSCs have been also involved in regulation of the monocyte/macrophage system. In particular, UC-MSCs can prevent the differentiation and maturation of monocytes toward DCs [194]. UC-MSCs also secrete other neuroprotective, angiogenic, and antiapoptotic factors, such as Neurotrophin 3 (NTF3), epidermal growth factor (EGF), neurite growth-promoting factor 2 (NEGF2/MDK), heparin binding EGF-like growth factor (HBEGF), Chemokine ligand 2 (CXCL2), CXCL5, and fibroblast growth factor 9 (FGF9) [195].

Angiogenesis/wound healing—Being mainly involved in WJ remodeling, UC-MSCs are not in contact with capillaries and small blood vessels, excluding the unique three vessels involved in umbilical blood circulation (the two arteries and the one vein), and they produce small amounts of angiogenic factor VEGF-A. Their support on endothelial cell proliferation and vasculogenesis could be related with other VEGF-independent factors, such as IL-8, hepatocyte growth factor (HGF), and MCP-1 [196].

Matrix remodeling—The UC-MSCs are involved in ECM composition of WJ that surrounds the umbilical vessels, expressing vimentin and collagen II [163]. Lo Iacono et al., demonstrated, using mass spectrometry analyses, that UC-MSCs co-cultured with umbilical cord blood–CD34+ hematopoietic stem/progenitor cells are able to secrete collagens, different proteases and their inhibitors, such as MMP-8, TIMP-1, ADAM with thrombospondin type 1 motif 9 (ADAMTS9), secreted protein acidic and cysteine rich (SPARC), plasminogen activator inhibitor-1 (PAI-1), and serine carboxypeptidase 1 involved in ECM remodeling, as well as α-2-HS glycoprotein, which is a TGF-β antagonist and prevents calcification by buffering excess matrix mineralization, in addition to than mimicking a hematopoietic niche [158]. Migration of cells on ECM, remodeling, and degradation of the ECM by MMPs are key regulators of wound repair, since wound healing requires the controlled activity of MMPs, and UC-MSCs may have a great potential in connective tissues and wounds [196,197,198].

Hypoxia resistance—Another aspect worthy of note about UC-MSCs is their high resistance to a stromal environment that is relatively hypoxic, therefore adapting to survive in limited nutrient and oxygen conditions [199]. To this regard, we recently demonstrated similar metabolism and survival capability in both normal and hypoxic conditions (in oxygen–glucose deprivation/reperfusion stroke model) exerted by the three different MSC populations isolated from the three different zones of umbilical stromal, Wharton’s jelly (WJ-MSCs), perivascular region (PV-MSCs), and cord lining (CL-MSCs) of human, suggesting that UC-MSCs are suitable for stem-cell-based therapy of ischemic diseases [200]. This provides the idea that tissue function support may be due to the transfer of healthy mitochondria [201], which has been demonstrated, improving oxidative phosphorylation (OXPHOS) and bioenergetics of recipient cells [202]. Moreover, our group demonstrated the secretion by UC-MSCs of the stress-induced chaperone hypoxia upregulated protein-1/GRP170, thought to have an important cytoprotective role in hypoxia-induced cellular perturbation [158].

The paracrine effects of UC-MSCs-derived molecules with immunomodulatory, anti-inflammatory, and regenerative properties are related to their release by MSCs on the extracellular environment not only through the secretion of soluble factors, but also as cargos of EVs, such as exosomes, which also contain lipids, metabolites, DNA fragments, miRNA fragments, and noncoding RNAs, acting locally and/or at distance as a cell-to-cell communication system, both in physiological and pathological conditions [203,204,205]. Due to their structure, and the opportunity to freely circulate into the body fluids, with low immunogenicity, and their bioavailability, they could be useful for drug delivery, and generate a great interest among scientists for cell-free therapies. The roles of perinatal MSCs and their conditioned media, enriched in EVs, were also explored in preventing lung injury across lung transplantation, described by Miceli and coworkers [206,207].

**Figure 3:**
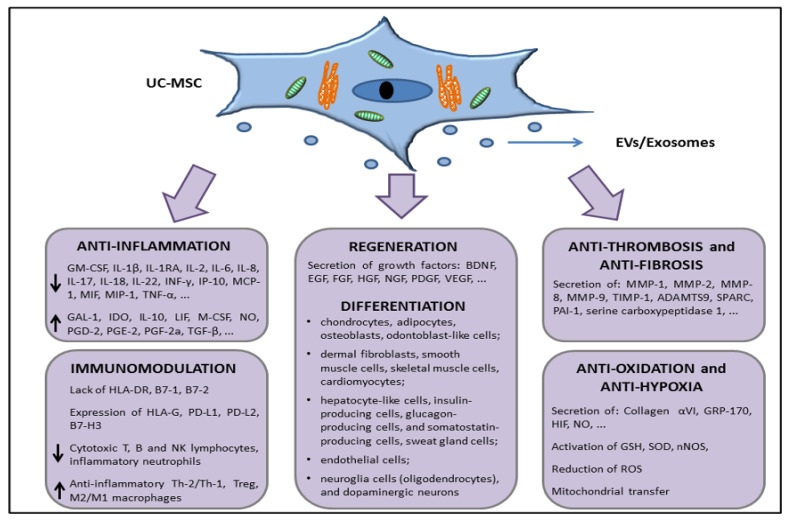
Schematic representation of the UC-MSCs effects related with molecules involved in direct cell-to-cell interaction or through the release of extracellular vesicles (EVs) and/or exosomes.

Taken together, all these features, characterized by the surface expression or secretion (through EVs/exosomes or in soluble form) of a series of factors with anti-inflammatory, immunomodulation, tissue repair, antifibrotic, and antihypoxic functions (summarized in Figure 3), support the interest in further studies about the use of UC-MSCs in in vivo experiments in order to move to allogeneic transplantation or develop novel cell-free products that are able to restore tissue functions, such as lung parenchymal functions dampening the damages exerted during COVID-19 disease.

### 5.3. In Vivo Preclinical Data Supporting the Use of UC-MSCs to Treat Organ Dysfunctions

The researchers worldwide have been focused on xenogeneic UC-MSCs in in vivo preclinical trials for determining safety, tolerance, and efficacy.

It was found that UC-MSCs transplantation induced, in vivo, a weak activation of immune Th1 and Th2 cells, and they showed significantly longer survival times in immunocompetent Balb/c mice compared to BM-MSCs, with a survival time prolonged in immunodeficient SCID-beige mice that must be attributed to the compromised immune response of the host [208].

Transplantation by intravenous (IV) injection in female dark agouti rats, used for EAE model, revealed that UC-MSCs were detected in lung and spleen 2.5 weeks after transplantation in the chronic disease phase, but they were not observed in the lymph nodes, spinal cord, or brain of transplanted animals. However, they potently (even if transiently) ameliorated neurological symptoms [209]. As explained above, multiple sclerosis could be alleviated by neuroprotective and neuroregenerating properties of LIF released in the bloodstream by UC-MSCs [190].

Studies of the pathogenesis of autoimmune type I diabetes demonstrated that UC-MSCs reduced systemic and pancreatic levels of cell populations, such as Th1 and Th17 cells, and a shift toward the Th2 profile along with an increase in Treg cells was found in UC-MSCs-treated nonobese diabetic (NOD) mice [189]. Moreover, in diabetic rats it was shown that UC-MSCs could survive both in the pancreas and in the liver with improved hyperglycemia due to the support of damaged insulin-producing β cells [210]. 

In a myocardial infarction murine model, induced by left anterior descending (LAD) coronary artery ligation, delivery of UC-MSCs by intramyocardial injection resulted in a reduction of scar in the left ventricular wall thickness and stimulation of angiogenesis, preventing apoptosis and attenuating adverse tissue remodeling, compared to the vehicle control group [211].

Focusing our attention on lung dysfunction, it was found that in an E. coli-induced pneumonia rat model, IV injection of fresh or cryopreserved CD362^+^ UC-MSCs, with or without concomitant antibiotic administration, revealed a decrease in the severity of lung injury, increased static lung compliance, significantly reduced bronchoalveolar lavage (BAL) bacterial load and inflammatory cytokine secretion, and increased airspace induced by CD362^+^ UC-MSCs compared to the vehicle. The efficacy of CD362^+^ UC-MSCs was not impaired by cryopreservation and the effects on arterial oxygen pressure were more expressed by the cell-treatment group compared to treatment with antibiotic or vehicle alone, but it was reduced along with the passages in culture of the cells and needed a rescue with a second dose of cells [212].

Although UC-MSCs do not differentiate into any lung cell, in the bleomycin-induced murine model of lung injury, these cells show homing to the lung tissue at 14 days after injection (but not at 28 days) compared to healthy mice, and showed antifibrotic properties increasing MMP-2, inducing the reduction of endogenous inhibitors (TIMP-1 to -4), and decreasing inflammation by repressing the expression of TGF-β, IFN-γ, and the proinflammatory cytokines macrophage migratory inhibitory factor (MIF) and TNF-α [213]. 

Even UC-MSCs-derived EVs have been under the microscope for treating organ dysfunction. In fact, as reviewed by Lelek and Zuba-Surma, in vivo preclinical studies on EVs derived from UC-MSCs have been conducted in neurological, cardiovascular, liver, kidney, and skin diseases [214]. In a preclinical model of bronchopulmonary dysplasia (BPD), using neonatal mice exposed to hyperoxia (75% O_2_), the IV injection with UC-MSCs-derived exosomes restored lung morphology, postnatal development, pulmonary hypertension, and vascular remodeling, along with decreased lung fibrosis [215]. Since the IV injection of MSCs resulted in entrapment of the cells in the lungs as first site after injection into the bloodstream [216], and taking into account what was already described, it was not surprising that researchers thought about the use of UC-MSCs for treating COVID-19 in clinical studies.

## 6. MSCs in COVID-19 Patients: Are UC-MSCs Better than the “Gold-Standard” BM-MSCs?

Based on just-reported evidence about the shorter survival time of BM-MSCs compared with UC-MSCs [208], there is still the consensus about the use of BM-MSCs as a “gold-standard” for cell therapy. This is because it is relatively safer to use autologous BM-MSCs (or AT-MSCs) from the same patients, compared to allogeneic UC-MSCs, but the age of the patients, their gender, their health conditions, and the invasive procedure for isolating BM-MSCs (or AT-MSCs) must be taken into account in order to balance pros and cons. Even BM-MSCs have been studied in COVID-19 patients, as reviewed in Yao et al. [217]. BM-MSCs are able to produce and secrete soluble PD-1 ligands (sPD-L1 and sPD-L2) that are responsible for hyporesponsiveness in T cells, arresting the PD-1-mediated AKT pathway, thus inducing immune tolerance [218]. Further, in in vitro and in a humanized mouse model of graft versus host disease (GvHD), it was described that BM-MSCs affected T lymphocyte proliferation more than UC-MSCs, while the latter induced a higher increase of Tregs/Th17 ratio [219]. Since Treg/Th17 ratio imbalance correlates with immune thrombocytopenia (ITP) [220] and this imbalanced ratio was also observed in different cases of COVID-19 disease [221], the reactions of infused MSCs for correcting immune response in such condition should be absolutely taken in consideration. For example, the patients with ITP displayed abnormalities in BM-MSCs, due to defects in mRNA and miRNA that induced downregulation of genes involved in cellular stress machinery, such as the unfolded protein response (UPR), the nuclear protein transcriptional regulator 1 (Nupr1), involved in endoplasmic reticulum pathway, the TGF-β1 signaling, leading to a loss of immunosuppressive properties, and a breakdown of self-tolerance in ITP patients [222]. In this specific case, ITP patients are not eligible for autologous BM-MSCs.

In a pilot study involving liver allograft recipients with acute rejection, they also observed a significant increase of Treg/Th17 ratio after 4 weeks of UC-MSC infusion [223]. Moreover, the BM-MSCs revealed a donor’s age-related decrease in colony forming units-fibroblast (CFU-f) in growth rate, in differentiation potential, and in superoxide dismutase (SOD) activity (and an increase of reactive oxygen species production) that could, in turn, affect autologous cell-based therapy [224]. On the contrary, UC-MSCs derived from perinatal tissue of childbearing age population showed no sign of senescence over several passages [157]. An integrated transcriptome-proteome analysis, comparing MSCs from different sources (BM, AT, and UC) revealed that secretome derived from UC-MSCs had a predominantly anti-inflammatory effect enriched in T cell inhibitory interleukins, such as IL-4, IL-13, IL-6, IL-35, IL-2, IL-22, IL-1R1, and IL-25, as well as the colony-stimulating growth factor (CSF) 3, which promoted M2 macrophage polarization, compared with the adult MSCs, while BM-MSCs were more immunosuppressive [225]. The immunosuppression is probably initiated starting from the apoptotic events induced by cytotoxic cells against BM-MSCs infused in GvHD recipients, as a result of a bystander effect of CD56^+^ natural killer (NK) and CD8^+^ T cells [226]. Taken together, these findings could highlight that UC-MSCs are more effective in treating symptoms and inflammatory state during the COVID-19-related cytokine storm than BM-MSCs.

## 7. Clinical Trials for the Treatment of COVID-19 Patients with UC-MSCs

The first paper on the use of MSCs (without a specific description of the cell source) for treating COVID-19 patients was published on 3 March 2020 in the journal “Aging and Disease” by Leng and colleagues, with a pilot study enrolling seven patients (and three for placebo control) from 23 January 2020 to 31 January 2020 (according to the guidance of National Health Commission of China), and demonstrating that after IV injection of 1 × 10^6^ cells/kg of weight, major symptoms (high fever, weakness, shortness of breath, and low oxygen saturation) disappeared in 2 to 4 days in all the patients, the oxygen saturations increased to ≥95% at rest, without or with oxygen uptake, the chest computer tomography (CT) scan showed that the ground-glass opacity (GGO) and pneumonia infiltration were reduced on the ninth day, there was a reduced amount of cytokine-secreting T cells, and two common and one severe patient were recovered and discharged in 10 days [227]. This study opened the path to further clinical trials.

Using the WHO International Clinical Trial Registry Platform (https://www.who.int/clinical-trials-registry-platform), accessed on 23 September 2022, 61 studies were documented in regards to the treatment of COVID-19 with UC-MSCs (or WJ-MSCs) and they are listed in Table 1.

As listed, the majority of the trials resulted in phase 1, but in some countries the UC-MSCs treatment for COVID-19 passed through phase 2 and only in Iran and Indonesia reached phase 3. Five clinical trials are testing the safety and efficacy of UC-MSCs- and WJ-MSCs-derived exosomes for the treatment of COVID-19 patients, and two of these trials are testing EVs even from other MSCs. Most of the clinical trials are ongoing, while 21 were completed, and the data were published for 15 of them [228,229,230,231,232,233,234,235,236,237,238,239,240,241,242,243,244,245], while 6 were case reports [246,247,248,249,250,251]. Results of these studies and their outcomes are listed and specified in Table 2.

Groups of patients enrolled for these trials were diagnosed with COVID-19-related ARDS, showing clinical symptoms from mild to critical, following the WHO severity classification described in the “Living guidance for clinical management of COVID-19” [15]. In particular, the clinical trials were mainly in early phases 1 to 2. Only one, a primary report of a two-center, open-label single-arm trial, arrived at a phase 2/3, using UC-MSCs or placental-derived MSCs [240]. The main administration route was a time-controlled IV infusion, while the pilot study, using exosomes, described an administration through nebulization [228], to easily reach the respiratory tract. Up to 210 patients were recruited and received IV infusion of 1–2 × 10^6^ UC-MSCs/kg in a single dose performed in several hospitals in Turkey [242]. In many cases, the dosage was repeated for two or three times (48-72 h after the first dose). In all the studies, no SAEs relative to UC-MSCs or WJ-MSCs-derived exosomes transplantation were observed (even with repeated infusions), supporting the safety of this treatment. The other visible ameliorations were related to the increased rate of survival and reduction of hospitalization times, improvement of all COVID-19 symptoms, including oxygen saturation that returned to normal ranges as shown by improved PaO_2_/FiO_2_ (partial pressure arterial of oxygen to fractional inspired oxygen) and SpO_2_/FiO_2_ (peripheral arterial oxygen saturation to FiO_2_), reduction of lung lesions and fibrosis, reduction of GGO, and mechanical ventilation need [231,232,234,235,236,237,238,240,242,243,245,246,247,248,249,250,251]. In the Monsel et al. study, the PaO_2_/FiO_2_ ratio change between day 0 and day 7 increased in the treated group, but it was not significant compared to the control group, and there were no differences in 28-day mortality [239]. However, a possible explanation of this contrasting result could be the small number of participants, the different doses of cells received (one, two, or three injections of 0.9 ± 0.1 × 10^6^ UC-MSCs/kg per dose) not considered in the final outcome, and the minor specific stratification based on the severity of their disease. Interestingly, Shu et al. also observed no progression from severe to critical illness with a faster restoration of symptoms of weakness, fatigue, shortness of breath, and low oxygen saturation after the treatment [231], together with significant decrease in CRP and IL-6 plasma level, normalization of lymphocyte count, and reduction of lung inflammation [232]. Reduced inflammatory state was determined by decreased levels of proinflammatory cytokines, such as IFN-γ, TNF-α, TNF-β, PDGF-BB, RANTES, sTNFR2, MCP-1, IP-10, IL-1RA, IL-5, IL-6, IL-8, IL-12, IL-17A, IL-17E/IL-25, IL-22, MIP-1, CXCL-1, reduction of CRP, D-dimer, ferritin, liver aminotransferase (GOT), MMPs, and an increase of IL-1β IL-10, LIF, SDF-1, KGF, and NGF [229,230,231,232,233,234,235,236,240,241,243,244,248,249,251]. Proangiogenic factor VEGF shows differences in plasma level when comparing different studies. For example, it increased in Dilogo et al. [230] and Adas et al. [243], but Saleh et al. [233] described a decreased level. The importance of VEGF expression in MSCs is linked to its biological action, in that VEGF is an angiogenic factor that promotes capillary regeneration and recovery of lung damage. Therefore, VEGF-expressing MSCs are viewed as a key factor protecting pulmonary vascular permeability [252]. On the other hand, a synergic action of hypoxia-induced angiogenic factors (VEGF, SDF-1, and ANG-II), hyperinflammation and cytokine storm, thrombosis, associated hemodynamic changes, and renin–angiotensin–aldosterone system dysregulation will trigger intussusceptive angiogenesis [253]. Moreover, COVID-19 patients showed high levels of SDF-1 associated with intussusceptive angiogenesis and T-lymphocytes infiltrates in lungs [64]. However, SDF-1 is also a key factor in facilitating MSCs homing and repair [254]. Nevertheless, the treatments with UC-MSCs resulted in normalization of immune cells infiltration with reduction of CD14^+^ monocytes, neutrophils, and NETs, and lymphocytes/neutrophils ratio, increase of CD3^+^, CD4^+^, CD8^+^, and Tregs [230,239,241,242,243,244,246], while NK cells were increased based on some studies [224,233] and decreased in another one [249]. In the case report published by da Silva et al., an increase of type-2 conventional dendritic cells (cDC2s) was found after UC-MSCs treatment [246]. This supports the immune modulating role of UC-MSCs, since it is known that type 1 DCs (DC1s) promote actions of CD8^+^, NK, and Th1 lymphocytes, while DC2s activate CD4^+^, Th1, Th2, Th17, and Treg lymphocytes [255]. Even the case report described by Senegaglia et al. showed a high absolute number of CD4^+^ and Treg, activated B-cells and plasmablasts (stimulating the production of specific SARS-CoV-2 antibodies), which is crucial in acute viral infections, but also a progressively decreased number of plasmablast at day 14 after cell treatment, demonstrating the patient’s recovery [251]. Even the use of the EVs alone, through nebulization, resulted in reduction of pulmonary lesions and a shorter hospitalization period in mild cases, and a twofold decrease was observed for the NK cells after exosome treatments [228].

Since UC-MSCs are HLA-G-expressing cells, they may contribute to modulate immune response in COVID-19 patients, even if the interaction with specific receptors in T cells, B cells, and macrophages (ILT-2, ILT-4, and KIR2DL4) in peripheral blood followed a high–low–high pattern, which may reflect the three subsequent stages of “infection”, “replication”, and “clearance” of SARS-CoV-2, as described in a single-patient study [256]. Further studies on HLA-G dynamics in a larger number of COVID-19 patients are necessary to better understand the potential role of this signaling cascade. UC-MSCs secrete LIF, which is able to directly oppose IL-6 activity and protect the lung function from injury [257]. Additionally, further studies are needed to better understand, for example, the pathways underlying VEGF and SDF-1 regulated by UC-MSCs to explain the contrasting functions of these molecules and to determine the meaning of some contradictory results found in the clinical trials.

Collectively, the use of UC-MSCs for treating patients affected by COVID-19, exhibiting ARDS-related symptoms and the risk of multiorgan failure have shown common evidence of amelioration of the critical signs that may lead to death, but limitations exist because of different severity of the illness of enrolled patients among the studies, and the concomitant use or not of other antiviral and/or inflammatory drugs, that could make it difficult to compare the results.

## 8. Discussion

The beginning of the zoonotic-related pandemic due to SARS-CoV-2 opened the door for the fragile healthcare system to face critical situations. The overload of the ICUs during COVID-19, the high rate of infectivity, the high number of deaths, and the lack of beds, led governments to declare lockdowns necessary worldwide, with all their economic and social consequences. SARS-CoV-2 will not be the last virus responsible for a pandemic, but what has been addressed during these last years has challenged the effectiveness of standard therapies, using well-known drugs. This aspect could be taken into account even to address both emerging and re-emerging infections. The beneficial properties of UC-MSCs (immunomodulation, immune modulatory, tissue repair, organ function recovery) exerted in COVID-19 patients may suggest their indication for the treatment of diseases which feature viral infection, inflammation/cytokine storm, and hypoxic microenvironmental conditions, present not only in lungs of patients infected by SARS-CoV-2, but also by other respiratory pathogens, or present in organs with failed functions due to other infectious diseases. Moreover, umbilical cord, even being one of the most MSCs-enriched tissues, is still considered a waste after childbirth, being therefore easily and painlessly obtained from young donors, weakening the concerns related to ethical issues. Nevertheless, it could be necessary to fill the gaps between the cure and the compliance with the regulations for the manufacture and clinical application of allogeneic UC-MSCs. In fact, UC-MSC-based products have been classified as advanced therapy medicinal products (ATMPs) since 2007, according to the European Regulation 1394/2007/EC [258]. Despite a series of completed clinical trials, only seven cord-blood-based cell therapies have received international approval from the Food and Drug Administration (FDA) [259]. However, UC-MSCs have been approved as interventional new drug (IND) in subjects with type 1 diabetes and Alzheimer’s disease [240]. In December 2014, the European Medicines Agency (EMA) recommended the first ATMP containing stem cells for approval in the European Union, regarding ex vivo expanded autologous human corneal epithelial cells containing stem cells [260]. Moreover, on 20 March 2017, orphan designation (EU/3/17/1852) was granted by the European Commission to Regulatory Resources Group Ltd., United Kingdom, for NiCord, allogeneic ex vivo-expanded umbilical-cord-blood-derived hematopoietic CD34^+^ progenitor cells and allogeneic nonexpanded umbilical-cord-blood-derived hematopoietic mature myeloid and lymphoid cells, for treatment in hematopoietic stem cell transplantation [261]. Up to date, most of the MSC-based ATMPs are composed of autologous BM-MSCs or AT-MSCs, and only a French manufacturing process of the UC-MSC-based ATMP was qualified and authorized by the French regulatory agency for clinical use [262]. However, even if all the findings reported in this review shed new light on the use of allogeneic UC-MSCs as ATMP or cell-free therapies (through EVs/exosomes), no UC-MSC-derived medicine has yet been authorized for marketing authorization by EMA. For these reasons, UC-MSC-based ATMP authorizations still need (i) to increase the number of studies related with safety and efficacy; (ii) to increase the number of participants enrolled in randomized controlled studies; (iii) to correctly stratify the patients; (iv) to standardize the dose of cell infused, the route of infusion, and the number of infusions at specific time points, following the long-term effects after the treatment; and (v) to standardize all the procedures regarding the investigational product, from cell isolation to the authorized final product, passing thorough the scale-up procedures in bioreactors under GMP regulations, and the choice of the best cell passage, all in order to reduce the discrepancies of outcomes between the trials, reduce contradictory results, and obtain authorizations. The development and manufacture of ATMPs are, at present, difficult, slow, and high in cost compared with production of traditional drugs, and UC-MSC-based ATMPs’ authorization, availability, assessment, and affordability are still the great challenges for both academic institutions and pharmaceutical companies.

## 9. Conclusions

It is now known that COVID-19 has become a global pandemic for which there is no specific cure for serious and/or critical patients. Initially, the clinical trials involved therapies with antiviral or immunomodulatory molecules, as well as neutralizing antibodies and immune plasma from convalescent patients for hospitalized patients with signs of ARDS and pathologies resulting from SARS-CoV-2. Nevertheless, these strategies resulted in poor outcomes and reproducibility. In this regard, MSCs could represent excellent candidates for their intrinsic immunomodulatory, anti-inflammatory, and reparative (antifibrosis and angiogenesis) properties, mediated through direct cell–cell interaction, the secretion of specific molecules, and the release of EVs. To date, encouraging results have been obtained from ongoing clinical trials involving the treatment of COVID-19 patients with adult and perinatal MSCs. However, it is important to underline that, compared to adult MSCs, perinatal MSCs possess a higher proliferative capacity with a lower risk of rejection, as well as ready availability through noninvasive procedures and without ethical issue. These are reasons that led us to discuss in this review the promising results obtained in therapies based on the infusion of UC-MSCs in patients affected with COVID-19. As we have observed, there are numerous advantages that can be acquired through the treatment with UC-MSCs or their derived EVs, such as the decrease in the levels of proinflammatory cytokines and the increase in the survival rate, combined with the robust safety and efficacy of the therapy. In conclusion, we can state that UC-MSCs could be a potent tool against phlogistic status, not only during the last pandemic caused by SARS-CoV-2, but also for other future pandemic or respiratory tract inflammatory diseases. However, a series of challenges still lies ahead, comprising the accomplishment of all the tests required by the regulatory bodies.

## Figures and Tables

**Table 1:** Clinical trials using MSCs derived from umbilical cord.

N°	Trial ID	Recruitment Status	Study Status	Treatment	Phase	Country
1	NCT04573270	Completed	Completed	UC-MSCs	1	USA
2	NCT04288102	Completed	Completed	UC-MSCs	2	China
3	NCT04625738	Completed	Completed	WJ-MSCs	2	France
4	NCT04355728	Completed	Completed	UC-MSCs	1–2	USA
5	NCT04392778	Completed	Completed	UC-MSCs	1–2	Turkey
6	NCT04400032	Completed	Completed	UC-MSCs	1–2	Canada
7	NCT04333368	Completed	Completed	WJ-MSCs	1–2	France
8	NCT04252118	Completed	Completed	UC-MSCs	1	China
9	NCT04457609	Completed	Completed	UC-MSCs	1	Indonesia
10	NCT05286255	Recruiting	Ongoing	UC-MSCs	1	USA
11	NCT04896853	Recruiting	Ongoing	WJ-MSCs	1	Sweden
12	NCT05387278	Recruiting	Ongoing	UC-MSCs and PL-derived exosomes	1	USA
13	NCT04869397	Recruiting	Ongoing	WJ-MSCs	2	Canada
14	NCT04865107	Recruiting	Ongoing	UC-MSCs	2	Canada
15	NCT04390139	Recruiting	Ongoing	WJ-MSCs	1–2	Spain
16	NCT04390152	Recruiting	Ongoing	WJ-MSCs	1–2	Colombia
17	NCT04494386	Recruiting	Ongoing	CL-MSCs	1–2	USA
18	NCT04399889	Recruiting	Ongoing	UC-MSCs	1–2	USA
19	NCT03042143	Recruiting	Ongoing	CD362 enriched UC-MSCs	1–2	UK
20	NCT05132972	Recruiting	Ongoing	UC-MSCs	2–3	Indonesia
21	NCT05240430	Recruiting	Ongoing	UC-MSCs	N/A	Turkey
22	NCT04313322	Recruiting	Unknown	WJ-MSCs	1	Jordan
23	NCT04437823	Recruiting	Unknown	UC-MSCs	2	Pakistan
24	NCT04269525	Recruiting	Unknown	UC-MSCs	2	China
25	NCT04339660	Recruiting	Unknown	UC-MSCs	1–2	China
26	NCT04371601	Not yet recruiting	Active, not recruiting	UC-MSCs	Early 1	China
27	NCT04456361	Not yet recruiting	Active, not recruiting	WJ-MSCs	Early 1	Mexico
28	NCT04452097	Not yet recruiting	Active, not recruiting	UC-MSCs	1–2	USA
29	NCT05501418	Not yet recruiting	Active, not recruiting	UC-MSCs	1–2	Taiwan
30	NCT04398303	Not yet recruiting	Unknown	UC-MSCs	1–2	USA
31	NCT04429763	Not yet recruiting	Unknown	UC-MSCs	2	Colombia
32	NCT04273646	Not yet recruiting	Unknown	UC-MSCs	N/A	China
33	EUCTR2020-002772-12	Completed	Completed	WJ-MSCs	2	France
34	EUCTR2020-001505-22	Recruiting	Ongoing	WJ-MSCs	1–2	Spain
35	EUCTR2020-001577-70	Recruiting	Ongoing	UC-MSCs and others MSCs	1–2	Italy
36	ChiCTR2000030173	Completed	Completed	UC-MSCs	Early 1	China
37	ChiCTR2000030088	Completed	Completed	WJ-MSCs	Early 1	China
38	ChiCTR2000030866	Completed	Completed	UC-MSCs	Early 1	China
39	ChiCTR2000030261	Completed	Completed	WJ-MSCs-derived exosomes	Early 1	China
40	ChiCTR2000030944	Completed	Completed	UC-MSCs	1	China
41	ChiCTR2000030138	Completed	Completed	UC-MSCs	2	China
42	ChiCTR2000031430	Completed	Completed	UC-MSCs	2	China
43	ChiCTR2000030116	Completed	Completed	UC-MSCs	N/A	China
44	ChiCTR2000030835	Completed	Completed	UC-MSCs	N/A	China
45	ChiCTR2000030484	Not yet recruiting	Active, not recruiting	UC-MSCs and exosomes	N/A	China
46	ChiCTR2000031494	Recruiting	Ongoing	UC-MSCs	1	China
47	IRCT20190717044241N2	Completed	Completed	WJ-MSCs	1	Iran
48	IRCT20200217046526N2	Completed	Completed	UC-MSCs	2–3	Iran
49	IRCT20190101042197N2	Completed	Unknown	UC-MSCs-derived exosomes	1–3	Iran
50	IRCT20201202049568N3	Completed	Unknown	UC-MSCs-derived exosomes	1–2	Iran
51	IRCT20160809029275N1	Completed	Unknown	UC-MSCs	2–3	Iran
52	IRCT20200421047150N1	Completed	Unknown	WJ-MSCs	2–3	Iran
53	IRCT20200426047206N2	Completed	Unknown	UC-MSCs	3	Iran
54	IRCT20140528017891N8	Completed	Unknown	UC-MSCs	3	Iran
55	IRCT20211012052743N1	Recruiting	Ongoing	UC-MSCs	3	Iran
56	JPRN-JapicCTI-205465	Recruiting	Ongoing	UC-MSCs	1	Japan
57	CTRI/2020/08/027043	Not yet recruiting	Unknown	UC-MSCs	1	India
58	CTRI/2021/09/036645	Recruiting	Ongoing	UC-MSCs	1–2	India
59	RBR-3fz9yr	Completed	Ongoing	UC-MSCs	N/A	Brazil
60	RBR-4jh63b	Not yet recruiting	Unknown	UC-MSCs	1–2	Brazil
61	RBR-8zg5rg7	Recruiting	Ongoing	UC-MSCs	1–2	Brazil

Recruitment status: Completed—the participants are no longer being examined or treated; Recruiting—the study is currently recruiting participants; Not yet recruiting—the study has not started recruiting participant. Study status: Completed—the study has ended normally; Ongoing—ongoing recruitment; Unknown—the status has not updated or verified within the past 2 years; Active, not recruiting: ongoing study but the recruitment has not started. N/A: trials without FDA-defined phases. MScs: mesenchymal stromal cells; UC-MSCs: umbilical cord MSCs; PL-derived: placental derived; CL-MSCs: cord lining-derived MSCs; WJ-MSCs: Wharton’s Jelly-derived MSCs.

**Table 2:** Results of published clinical studies based of UC-MSCs and WJ-MSCs (or derived exosomes) therapy administered to COVID-19 patients.

Type of Study	Phase	Number of Patients	COVID Symptoms	Treatment	Outcomes	Ref.
Pilot trial	Early 1	7	Mild 5 Severe 2	Nebulization ranged from 7.66 × 10^0.8^ to 7.00 × 10^0.7^ WJ-MSCs-derived exosomes/mL, twice a day, up to discharge.	Reduction of pulmonary lesions and period of hospitalization in mild cases and reduction in cellular residue in severe cases. No adverse events were observed.	Chu et al., 2022 [228]
Parallel assigned controlled, nonrandomized trial	1	18	Moderate 10 Severe 8	Moderate = 5; Severe = 4; Infusion of 3 × 10^7^ UC-MSCs for 3 times on days 0, 3, and 6.	Reduced trend in plasma levels of inflammatory cytokines IFN-γ, TNF-α, MCP-1, IP-10, IL-1RA, IL-6, IL-8, IL-18, IL-22 and MIP-1. No serious adverse events were observed.	Meng et al., 2020 [229]
Double-blind, multicenter, randomized controlled trial	1	40	Critical	N = 20 patients; Infusion of 1 × 10^6^ UC-MSCs/kg in single dose.	Increased survival rate. Decrease trend in IL-6 levels and increase trend in IL-10, LIF and VEGF levels in plasma. No adverse events were observed.	Dilogo et al., 2021 [230]
Single-center open-label, individually randomized, standard treatment-controlled trial	1	41	Severe	N = 12 patients; Infusion of 2 × 10^6^ UC-MSCs/kg in single dose.	No progression from severe to critical illness. Reduction of weakness, fatigue, shortness of breath, and low oxygen saturation. Significant decreased in CRP and IL-6 plasma levels. Faster normalization in lymphocyte count and reduction of lung inflammation. No adverse events were observed. A 3-month follow-up of 28 patients (treated = 8, control = 20) revealed reduction of partial pulmonary function recovery time, ameliorated HRQL, and no adverse events were observed after 3 months.	Shu et al., 2020 [231] and Feng et al., 2021 [232]
Open-label, single-center trial	1	5	Severe	Injection of 150 × 10^6^ WJ-MSCs for 3 times on days 0, 3, and 6.	Increase in IL-10 and SDF-1 and decrease of VEGF, TGF-β, IFN-γ, IL-6, and TNF-α plasma levels. Improvement in hematology, myocardial enzyme, inflammation, and biochemical tests. No adverse events were observed.	Saleh et al., 2021 [233]
Single-center, open-label, placebo-controlled trial	1	20	Mild-to-moderate	N = 10 patients; Infusion of 1 × 10^6^ UC-MSCs/kg for 3 times on days 1, 3 and 5.	Significant improvement in SpO_2_/FiO_2_ ratio. Significant reduction in cytokine IL-6, IFN-γ, TNF-α, IL-17 A, and CRP levels and increase in cytokine levels of TGF-β, IL-1B, and IL-10. No serious adverse events were observed.	Kaffash Farkhad et al., 2022 [234]
Single-arm, pilot trial	2	16	Severe 9 Critical 7	Infusion of 1 × 10^8^ UC-MSCs for 4 rounds of transplantation.	Amelioration of oxygenation index. Increase of CD4+ T, CD8+ T, and NK lymphocytes. IL-2, IL-4, IL-6, IL-10, TNF-α, IFN-γ, and CRP have returned in the normal range. No adverse events were observed.	Feng et al., 2020 [235]
Single-blind, randomized, placebo-controlled trial	2	58	Mild 31 Severe 21 Critical 6	N = 29 patients; Mild 15 Severe 11 Critical 3 Infusion of 1 × 10^6^ UC-MSCs/kg in single dose.	Shorter hospital stay and symptoms remission. Improved CT scans. Reduction of CD14+ monocytes, CRP, NETs and proinflammatory cytokines IL-1RA, IL-18, IL-27, IL-17E/IL-25, IL-17F, GRO-alpha (CXCL-1), and IL-5. High expression of genes involved in chemotaxis, telomerase assembly and maturation, angiopoiesis, HSCs mobilization, and fetal extramedullary hematopoiesis, including VNN2. Stimulation of SARS-CoV-2 specific antibodies production. No adverse events were observed.	Zhu et al., 2021 [236]
Double-blind, randomized, placebo-controlled trial	2	100	Severe	N = 65 patients; Infusion of 4 × 10^7^ UC-MSCs for 3 times on days 0, 3, and 6.	Improvement in lung lesion volume, improved restoration of the integrated reserve capability, and normal CT scans after 1 year. Similar incidence of adverse events and tumor markers to placebo group after 1 year.	Shi et al., 2021 [237] and Shi et al., 2022 [238]
Multicenter, double-blind, randomized, placebo-controlled trial	2	45	Mild 31.1%; Moderate 48.9%; Severe 20%	N = 21 patients; Infusion of 0.9 ± 0.1 × 10^6^ UC-MSCs/kg per dose over 5 days (on day 1, day 3 ± 1, and day 5 ± 1) N = 17 − 3 doses N = 2 − 2 doses N = 2 − 1 dose.	UC-MSC-treated patients’ greater PaO_2_/FiO_2_-ratio increased between D0 and D7, with lack of statistically significant differences, compared to controls. No adverse events were observed.	Monsel et al., 2022 [239]
Double-blind, randomized, controlled trial	1–2	24	Mild-to-moderate 6; Moderate-to-severe 18	N = 12 patients; Mild-to-moderate 3 Moderate-to-severe 9 Infusion of 100 ± 20 × 10^6^ UC-MSCs for 2 times on days 0 and 3.	Significantly improved SAE-free survival and time to recovery. Significant reduction in plasma levels of inflammatory cytokines, chemokines, and growth factors GM-CSF, IFN-γ, IL-5, IL-6, IL-7, TNFα, TNF-β, PDGF-BB, RANTES, and sTNFR2. No difference in adverse events among both groups.	Lanzoni et al., 2021 [240] and Kouroupis et al., 2021 [241]
Randomized trial	1–2	210	Severe 111; Critical 99	Infusion of 1–2 × 10^6^ UC-MSCs/kg in single dose.	After 2–3 weeks after transplantation, improvement oxygen saturation and high survival rate, especially before intubation. No adverse events observed.	O Ercelen et al., 2021 [242]
Prospective double controlled trial	1–2	30	Moderate 10; Critical 20	N = 10 critical patients; Infusion of 3 × 10^6^ WJ-MSCs/kg for 3 times on days 0, 3, and 6.	Decrease in proinflammatory and profibrotic factors IL-6, CRP, IFNγ, IL-2, IL-12, IL-17A, MMP-9, and MMP-3 plasma levels. Increase in anti-inflammatory and angiogenesis promoting factors IL-10, TGF-β, VEGF, KGF, and NGF plasma levels. Reduction of the mechanical ventilation period and high survival rate. No adverse events were observed.	Adas et al., 2021 [243]
Prospective, single-center, randomized, double-blind, placebo-controlled trial	1–2	17	Critical	N = 11 patients; Infusion of 5 × 10^5^ UC-MSCs/kg every 48 h for 3 times.	Decrease in ferritin, IL-6 and MCP-1-CCL2, CRP, D-dimer, and neutrophils levels and reduction of lung damage. Increase in the numbers of lymphocytes T CD3+, CD4+ and NK. All these values are maintained until 4 months. No serious adverse events were observed.	Rebelatto et al., 2022 [244]
Primary report of a two-center, open-label, single-arm trial	2–3	11	Critical	Infusion of 200 × 10^6^ UC-MSCs (N = 6) and PL-MSCs (N = 5) every other day for 3 times.	Increased of SpO_2_. Significant reductions in serum levels of TNF-α, IL-8, and CRP. No adverse events were observed.	Hashemian et al., 2021 [245]
Case report	N/A	1	Severe	Infusion of 5 × 10^7^ UC-MSCs for 2 times on days 30 and 32.	Increased PaO_2_/FiO_2_ ratio. Decrease of inflammatory monocytes and increase of patrolling monocytes, CD4+ T lymphocytes, and cDC2. Reduction of lung infiltrates and fibrosis. No adverse events were observed.	da Silva et al., 2021 [246]
Case report	N/A	1	Critical	Infusion of 1 × 10^6^ UC-MSCs/kg in single dose.	Increase of SpO_2_ and absolute number of the lymphocytes. Reduction of GGO, lung infiltration, and plasma levels of CRP and D-dimer. No adverse events were observed.	Zhu et al., 2020 [247]
Case report	N/A	1	Severe	Infusion of 1 × 10^6^ WJ-MSCs/kg in single dose.	Reduction of GGO, lung infiltration and plasma levels of CRP, IL-6 and TNF-α. Increase of CD3+, CD4+, and CD8+ T lymphocytes. No adverse events were observed.	Zhang Y. et al., 2020 [248]
Case report	N/A	1	Critical	Infusion of 1 × 10^6^ UC-MSCs/kg for 8 times divided in 3 rounds.	Reduction of fiber strands and GGO. Reduction in IL-6, IL-10, WBCs, CRP, and D-dimer levels. Increase of CD4+ T lymphocytes and decrease of NK cells. No adverse events were observed.	Zhang Q. et al., 2021 [249]
Case report	N/A	1	Critical	Infusion of 5 × 10^7^ UC-MSCs for 3 times on days 13, 16, and 19.	Reduction of GGO, D-dimer, WBCs, neutrophils, and lymphocytes/neutrophils ratio. Increase of CD3+, CD4+, and CD8+ T lymphocytes. No adverse events were observed.	Liang et al., 2020 [250]
Case report	N/A	1	Severe	Infusion of 0.5 × 10^6^ UC-MSCs/kg for 3 times.	Reduction of GGO, plasmablasts, creatinine, GOT, ferritin, D-dimer, and CRP levels. Increase in the absolute number of total lymphocytes, CD4+ T, and Treg lymphocytes. No adverse events were observed.	Senegaglia et al., 2021 [251]

CRP: C-reactive protein; CT: computed tomography; GGO: ground-glass opacity; HRQL: health-related quality of life; HSCs: hematopoietic stem cells; NETs: neutrophil extracellular traps; PaO_2_/FiO_2_: partial pressure arterial of oxygen to fractional inspired oxygen; PL-MSCs: placental MSCs; SAE: serious adverse event; SDF-1: stromal cell-derived growth factor 1; KGF: keratinocyte growth factor; NGF: nerve growth factor; SpO_2_/FiO_2_: peripheral arterial oxygen saturation to FiO_2_; GOT: glutamic-oxaloacetic transaminase; VNN2: vascular noninflammatory molecule 2; WBCs: white blood cells.

## Data Availability

Not applicable.
